# The Effect of Stand Age on Throughfall Chemistry in Spruce Stands in the Potok Dupniański Catchment in the Silesian Beskid Mountains, Southern Poland

**DOI:** 10.1100/tsw.2007.90

**Published:** 2007-03-21

**Authors:** Stanisław Małek, Aleksander Astel

**Affiliations:** ^1^ Department of Forest Ecology, Forest Faculty, Agricultural University of Cracow, Al. 29 Listopada 46, 31–425 Krakêw, Poland; ^2^ Environmental Chemistry Research Unit, Biology and Environmental Protection Institute, Pomeranian Academy, 22a Arciszewskiego Str., 76-200 Słupsk, Poland

**Keywords:** bulk precipitation, throughfall, Norway spruce stands

## Abstract

The chemical composition of throughfall depends on the age of the Norway spruce (Picea abies Karst) stands and season of the year. The pH of throughfall decreased and the amount of hydrogen ion in throughfall deposited to the soil increased with increasing age of spruce stands, especially in the winter season. Concentrations of K^+^, H^+^, SO_4_^2−^, Mn^2+^, and NH^4+^ in throughfall were higher than bulk precipitation for the whole year and K^+^, H^+^, and Mn^2+^ concentrations were higher in throughfall in winter and the growing season. This indicates that these ions were washed out or washed from the surface of needles and/or the bark, and that NO_3_^−^, NH^4+^, Ca^2+^, Mg^2+^, Fe^2+^, and Zn^2+^ were absorbed in the canopy. The effect of high nitrogen deposition, above critical loads, and an increase in the amount of sulfur and in the sum of the strong acids (S-SO_4_^2−^ and N-NO_3_^−^) that reached the soil with throughfall may have implications for the vitality of spruce stands, especially in older age classes. The application of Principal Component Analysis (PCA) has led to identification of five factors responsible for the data structure (“mineral dust”, “acidic emissions”, “heavy metals-dust particles”, “ammonium [NH_4_^+^]”, and “H^+^”). They explain more than 60% of the total variance system. The strong positive correlation between stand age class and ionic concentrations in throughfall occurs for all year and the winter period for ions within the following categories: “acidic emissions”, SO_4_^2−^ + NO_3_^−^; “heavy metals-dust particles”, Fe^2+^ + Mn^2+^ + Zn^2+^; “mineral dust”, Na^+^ + K^+^ + Ca^+2^ + Mg^2+^; “NH_4_^+^”; and “H^+^”. The strength of the relationship decreases in the growing period, probably due to processes occurring in the canopy (adsorption, leaching, etc.).

## INTRODUCTION

Cycling of elements in Norway spruce (*Picea abies* Karst) stands, which are affected by industrial emissions, is still a subject of active research. The retention of considerable levels of contaminants by the canopy, and their removal or washout from needles by rainfall cause changes in the concentration of anions and cations reaching the soil surface[1]. Earlier investigations were based on relatively small forest catchments, such as Brenna[2,3,4], and on larger catchments, such as the Black and the White Vistula[5]. The goal of those studies was to estimate the volume and quality of the deposition of elements and their outflow from the catchment. Several case studies comparing elemental turnover in beech and spruce stands documented that, in general, deposition input to soil and the leaching of sulfur, nitrogen, and protons are higher in spruce stands[4]. Those studies did not usually take into account different development stages, either of stands or their spatial distribution in catchments. One study considered the effect of various development stages of spruce stands[1] and another one considered two different spruce age classes[6].

The present study examines the effect of different age classes (11–116 years) on throughfall chemistry in spruce stands in the Potok Dupniański catchment in the Silesian Beskid in the years 1999–2003. The objectives of this research were to determine: (1) if the chemical composition of throughfall water depends on the age of the spruce stands and season of the year, and (2) if the emission sources of the group of pollutants measured in the bulk precipitation (BP) and throughfall samples can be identified.

## METHODOLOGY, ANALYTICAL AND STATISTICAL TECHNIQUES

The Potok Dupniański catchment, 1.68 $km^2$ in area, is located in southern Poland in the Silesian Beskid Mts. (49°34'N, 18°50'E), not far from main industrial centers. This region of the Polish part of the Carpathian Mountains is under their negative effect[7,8]. The catchment is covered with Norway spruce (*P. abies* Karst) stands of different ages, growing on dystric cambisols developed on Istebna sandstone. The equipment for measuring throughfall in pure spruce stands (one monitoring plot in each age class) was set up in 1998. The 1st, 2nd, 5th, and 6th age classes were 11, 24, 91, and 116 years old, respectively, in 1999 (only one age class was found in each catchment). The descriptive characteristics of the spruce stands are presented in Table 1. A BP sampler was installed in the middle of the catchment at an elevation of 725 m a.s.l, close to the throughfall sampling point (within 500 m). The studies were conducted in 1999–2003 following methods described in the ICP-Forest Manual (1998)[9] and by Małek[1].

During the vegetation season, from May 1st to October 30th (the same year), samples of bulk precipitation directly reaching the catchment were collected from special collectors (five units with 15-cm inlet diameter each) installed in an open area 0.5 m above ground level, connected to a plastic tube with an outlet joining a container and a measuring device installed in a bunker. In winter, from November 1st (the previous year) to April 30th (the following year), six collectors (plastic, chemically neutral snow bags with 15-cm inlet diameter each) were installed at 1.3 m above ground level in the open area at a distance of 120–150 m from the forest edge. In order to evaluate the volume and quality of throughfall, water was sampled from a sampling system (this time, the number of collectors was 15, each with a 15-cm inlet diameter) installed under the canopy, similar to the one installed in the open area during the vegetation season. In winter, six collectors (plastic, chemically neutral snow bags with 15-cm inlet diameter each) were installed at 1.3 m above ground level in spruce stands of different age classes (described in detail by Małek[1]).

The sampling was performed on the 1st day of each month. Water was analyzed using ion chromatography (Dionex-320) to determine the concentration of $Cl^-$, $NO_3^-$, $SO_4^{2-}$, $F^-$, $NH_4^+$, $Na^+$, $K^+$, $Ca^{2+}$, $Mg^{2+}$, $Fe^{2+}$, $Mn^{2+}$, and $Zn^{2+}$. A low-pH acid rain sample from southern Ontario (Canada), RAIN.97 - No 409, served as a certified reference material (CRM) and was analyzed as well. When the concentration of analytes was below the limit of detection (LOD) of the analytical technique, the value of one-third LOD was used in the dataset due to chemometric requirements[10]. The limit of detection, expressed as

**TABLE 1**
**Descriptive Characteristics of Norway Spruce (*P. abies* Karst) Stands with Throughfall Plots in the Dupniański Stream Catchment**

| Kind of Plot | Age (year) | Elevation (m a.s.l.) | Diameter ($m^{-2}$) | Height (m) | Number of trees (no/1 ha) | LAI (Leaf Area Index) |
|---|---|---|---|---|---|---|
| | | | **1999** | | | |
| 1st age class | 11 | 720 | 2.2 | 1.5 | 20,150 | 5.0 |
| 2nd age class | 24 | 700 | 11.7 | 12.6 | 2,611 | 5.3 |
| 5th age class | 91 | 660 | 39.7 | 37.8 | 382 | 5.4 |
| 6th age class | 116 | 700 | 42.1 | 36.6 | 414 | 5.5 |
| | | | **2003** | | | |
| 1st age class | 16 | | 3.6 | 5 | 13,758 | 5.1 |
| 2nd age class | 29 | | 14.7 | 16.2 | 2,070 | 5.3 |
| 5th age class | 96 | | 42.1 | 39.1 | 350 | 5.4 |
| 6th age class | 121 | | 45.5 | 38.8 | 330 | 5.5 |

concentration or quantity, is derived from the smallest quantity that can be detected with reasonable certainty for a given procedure[11]. Replacement values were used in 1, 3, 29, and 16 samples used for the determination of $K^+$, $Mg^{2+}$, $Zn^{2+}$, $Mn^{2+}$, respectively. In the dataset, there were two variables with significantly high percentage of measurements below LOD ($Fe^{2+}$, $F^-$). One of the ions ($Fe^{2+}$ - 25% measurements below LOD) was subjected to chemometrical evaluation mainly due to the small value of the mean and the median. Data for $F^-$ were excluded from the analyses.

A commercial statistics software package, Statistica 6.0 for Windows, was used for chemometric data mining[12]. Before chemometrical evaluation, the data were normalized by logarithmic transformation in the form x' = log(x) for ion concentration, due to considerable asymmetry of analyte concentration values. Analysis of variance was performed on logarithmic concentration values after dividing the whole set into two additional subsets concerning winter (from November to April) and the vegetation season (from May to October). Moreover, autoscaling was performed prior to Principal Component Analysis (PCA) evaluation. The validation of the obtained PC models was performed by consideration of the scree plot (empirical testing) and only those PCs were included in the model, which possess eigenvalues greater than or close to 1. Significance of the component model retained was additionally tested by applying Bartlett's statistics[13] and the model with three components was selected for the 5th forest stand age class category, four PCs for BP, and five PCs for the 1st, 2nd, and 6th forest age class categories, which in general confirms scree plot analysis results[13]. Factors obtained by PCA were rotated using the orthogonal varimax rotation.

## RESULTS AND DISCUSSION

After passing through the canopy (Table 2), water decreased its pH value: in winter – from 5.09 (in BP) to 4.97 (stands of 1st age class), 4.60 (2nd age class), 4.21 (5th age class), and 4.11 (6th age class); in the growing season – from 5.25 (in BP) to 5.04 (1st age class), 4.99 (2nd age class), 4.42 (5th age class), and 4.41 (6th age class), indicating that decreasing pH and an increasing amount of hydrogen ion was deposited to the soil in throughfall with increasing stand age, especially in the winter season.

**TABLE 2**
**Five-Year Average (1999–2003) Deposition Fluxes in BP and Throughfall Water in Different Norway Spruce (*P. abies* Karst) Stand Age Classes (1st, 2nd, 5th, and 6th)* (in kg/ha) and pH of Water in the Dupniański Stream Catchment in Particular Seasons**

| Kind of Water | V (mm) | $Cl^-$ | $N\text{-}NO_3^-$ | $N\text{-}NH_4^+$ | $S\text{-}SO_4^{2-}$ | $Na^+$ | $K^+$ | $Ca^{2+}$ | $Mg^{2+}$ | $Fe^{2+}$ | $Mn^{2+}$ | $Zn^{2+}$ | $H^+$ ($g\ ha^{-1}$) | pH |
|---|---|---|---|---|---|---|---|---|---|---|---|---|---|---|
| | | ($kg\ ha^{-1}$) | | | | | | | | | | | | |
| | | | | | | **Whole Year** | | | | | | | | |
| BP | 1,072 | 19.70 | 9.02 | 12.24 | 13.67 | 7.67 | 9.17 | 32.73 | 4.41 | 0.52 | 0.17 | 2.34 | 70.82 | 5.18 |
| 1st | 933 | 9.27 | 5.80 | 9.26 | 10.98 | 4.14 | 11.14 | 16.13 | 2.09 | 0.44 | 0.45 | 0.30 | 92.75 | 5.00 |
| 2nd | 833 | 14.08 | 8.53 | 8.30 | 15.99 | 5.43 | 19.59 | 17.79 | 2.24 | 0.49 | 0.62 | 0.33 | 148.28 | 4.75 |
| 5th | 696 | 10.25 | 6.84 | 9.61 | 17.54 | 3.22 | 16.00 | 19.12 | 2.71 | 0.34 | 1.98 | 0.41 | 332.30 | 4.32 |
| 6th | 624 | 10.96 | 6.78 | 9.50 | 17.28 | 3.38 | 12.87 | 20.96 | 3.04 | 0.41 | 0.88 | 0.43 | 342.00 | 4.26 |
| | | | | | | **Growing Season** | | | | | | | | |
| BP | 639 | 14.43 | 6.45 | 7.67 | 8.60 | 3.71 | 6.93 | 22.87 | 2.79 | 0.39 | 0.11 | 2.15 | 35.56 | 5.25 |
| 1st | 475 | 4.41 | 3.31 | 5.11 | 6.88 | 2.12 | 9.47 | 9.53 | 1.26 | 0.25 | 0.35 | 0.17 | 43.48 | 5.04 |
| 2nd | 412 | 7.73 | 5.07 | 4.95 | 7.75 | 3.41 | 15.10 | 9.37 | 1.11 | 0.27 | 0.38 | 0.12 | 41.71 | 4.99 |
| 5th | 399 | 4.99 | 4.11 | 5.86 | 8.93 | 1.50 | 10.68 | 10.03 | 1.23 | 0.22 | 0.91 | 0.23 | 150.72 | 4.42 |
| 6th | 364 | 4.92 | 3.52 | 5.82 | 8.69 | 1.51 | 8.52 | 11.20 | 1.64 | 0.25 | 0.38 | 0.21 | 140.52 | 4.41 |
| | | | | | | **Winter Season** | | | | | | | | |
| BP | 432 | 5.27 | 2.57 | 4.56 | 5.07 | 3.96 | 2.24 | 9.87 | 1.62 | 0.13 | 0.07 | 0.19 | 35.26 | 5.09 |
| 1st | 457 | 4.87 | 2.50 | 4.15 | 4.10 | 2.02 | 1.68 | 6.60 | 0.83 | 0.19 | 0,10 | 0.13 | 49.27 | 4.97 |
| 2nd | 421 | 6.35 | 3.46 | 3.35 | 8.24 | 2.01 | 4.48 | 8.42 | 1.13 | 0.22 | 0.25 | 0.20 | 106.57 | 4.60 |
| 5th | 297 | 5.27 | 2.72 | 3.76 | 8.61 | 1.72 | 5.32 | 9.09 | 1.48 | 0.12 | 1.07 | 0.18 | 181.59 | 4.21 |
| 6th | 260 | 6.05 | 3.27 | 3.67 | 8.59 | 1.87 | 4.36 | 9.76 | 1.40 | 0.16 | 0.51 | 0.22 | 201.49 | 4.11 |

* The four stand age categories correspond to stands that are 11, 24, 91, and 116 years old.

Concentrations of $K^+$, $H^+$, $SO_4^{2-}$, $Mn^{2+}$, and $NH_4^+$ in throughfall were higher than BP for the whole year, and $K^+$, $H^+$, and $Mn^{2+}$ concentrations were higher in throughfall in winter and the growing season. These results indicate that these ions were washed out or washed from the surface of needles and/or the bark. The other ions, $NO_3^-$, $NH_4^+$, $Ca^{2+}$, $Mg^{2+}$ (especially in vegetation period), $Fe^{2+}$, and $Zn^{2+}$, were absorbed by the canopy (Table 2, Figs. 1 and 2). A substantial absorption of nitrate, especially during the growing season, confirms the results of Lovett at al.[14]. In the stands of the 2nd age class, up to 80% of nitrogen was absorbed in tree crowns, a value similar to the one established by Zimka and Stachurski[15].

Tree crowns, and especially needle surfaces, are places where dust pollutants, particularly heavy metals, such as Zn and Cd, are deposited[16]. These pollutants are removed from canopies by precipitation. The effectiveness of their removal differs according to the ion and tree species[17]. It also depends on other factors, namely the presence of $NH_4^+$ ions and epiphytes, the concentration of elements in the foliage, and on ion exchange reactions. These reactions make it possible for plants to take up elements directly from rainwater, which causes the removal of $Na^+$, $K^+$, and $Zn^{2+}$ from the plants, thus intensifying the transfer of those ions to the soil[18]. In the case of spruce stands growing in the Potok Dupniański catchment, concentrations of $K^+$ and $Mn^{2+}$ in throughfall were higher than in BP for all stand ages, as in the year 2000[1]. Magnesium and calcium are probably absorbed directly from rainfall, possibly compensating for the considerable washout of these elements beyond the reach of the root system[5].

The effect of nitrogen deposition may be an increasing problem in the future unless emissions of nitrogen oxides and especially of $NH_4^+$ are reduced[19]. Five-year average bulk deposition to the Dupniański catchment in kg/ha/year of sulfur ($S\text{-}SO_4^{2-}$) was 13.7, whereas in throughfall in each particular spruce age class, it was, respectively, 1st age class, 11.0; 2nd age class, 16.0; 5th age class, 17.5; and 6th age class, 17.3; indicating an increasing amount of sulfur with the spruce age especially in winter

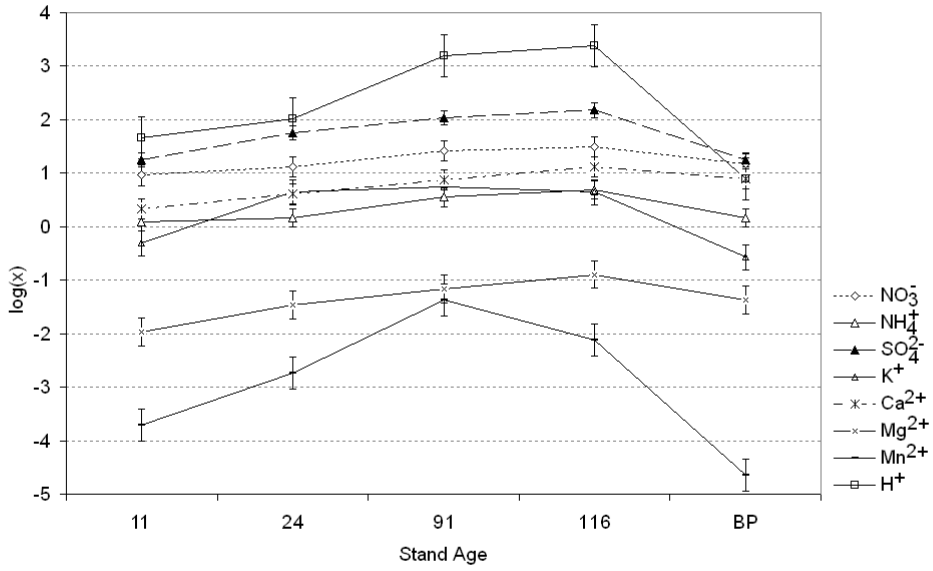

**FIGURE 1.** Distribution of logarithms of mean concentrations of $NO_3^-$, $NH_4^+$, $SO_4^{2-}$, $K^+$, $Ca^{2+}$, $Mg^{2+}$, $Mn^{2+}$ (all ions in mg·l$^{-1}$), and $H^+$ (µg·l$^{-1}$) in throughfall for four Norway spruce (*P. abies* Karst) forest stand categories and in BP for the whole year (vertical lines represent confidence intervals, $p$ = 0.05).

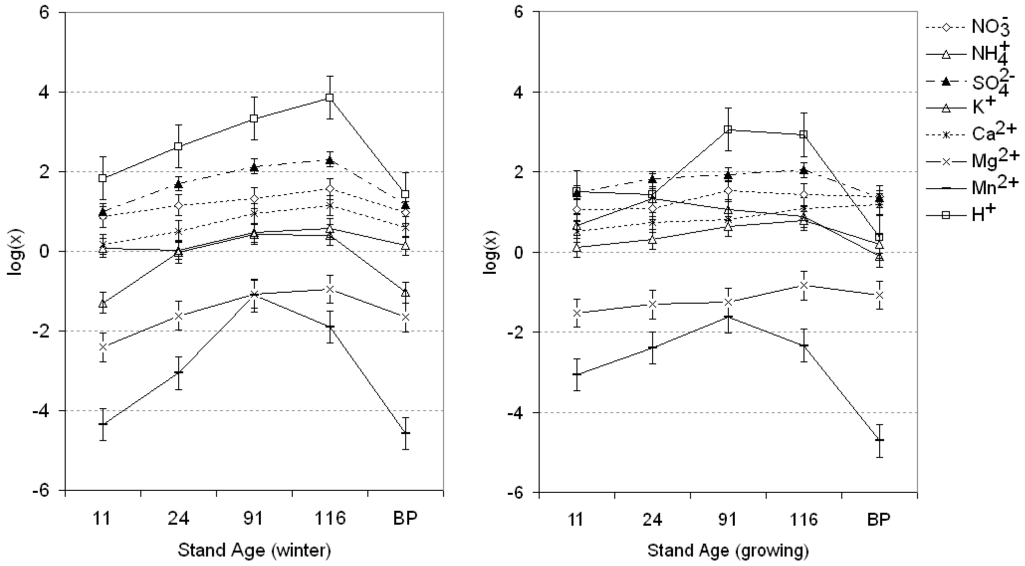

**FIGURE 2.** Distribution of logarithms of mean concentrations of $NO_3^-$, $NH_4^+$, $SO_4^{2-}$, $K^+$, $Ca^{2+}$, $Mg^{2+}$, $Mn^{2+}$ (all ions in mg·l$^{-1}$), and $H^+$ (µg·l$^{-1}$) in throughfall for four Norway spruce (*P. abies* Karst) forest stand categories, stand age categories, and in BP in winter and in the growing season (vertical lines represent confidence intervals, $p$ = 0.05).

period. The sum of the strong acids (S-$SO_4^{2-}$ and N-$NO_3^-$) in bulk deposition was around 22.7 kg/ha/year, but in throughfall was around 24.5 in older spruce age classes (Table 2). Nitrogen deposition (sum N-$NO_3^-$ and N-$NH_4^+$) was above the critical load (15–20 kg N/ha/year) for coniferous trees, which may change N/macronutrients ratios, decrease K and Mg, and increase N concentration in foliar tissue[20]. Nitrogen deposition fluxes in this study were below the values reported for the heavily polluted Ore Mts. (Czech Republic)[21].

Despite large emission reductions, the Dupniański Stream catchment still suffers from very high loads of acidifying input deposited during the past decades. Soil recovery depends on future emissions, especially on base cation deposition. The recovery will be even slower if base cation deposition decreases further[22]. The soil in the Dupniański catchment is very acidic and has relatively small pools of exchangeable $Ca^{2+}$ and $Mg^{2+}$. The trees show medium to severe nutrient deficiency symptoms, such as needle loss and needle yellowing[23]. Soil solution concentrations of $SO_4^{2-}$, $Ca^{2+}$, and $Mg^{2+}$ have generally decreased, while pH values remained stable throughout the study period, similar to that reported for Fichtelgebirge-NE-Bavaria, Germany[24]. Decreasing concentrations of cations in soil solution occurred especially in the growing period, which can affect nutrient cycling and mineral nutrition. This is of particular concern because magnesium is necessary for the proper development of plants (and whose deficit was observed in the same spruce stands in earlier studies[23]), along with the accumulation of heavy metals. The relatively low pH in throughfall may contribute to the release of heavy metals in the sorption complex of soil, which can adversely affect the development and health of spruce stands.

Ionic concentrations in BP and throughfall with varying stand ages are presented in Figs. 1 and 2 for $NO^{3-}$, $NH_4^+$, $SO_4^{2-}$, $K^+$, $Ca^{2+}$, $Mg^{2+}$, $Mn^{2+}$, and $H^+$. The lines connecting the mean value points were drawn for illustrative purposes only and do not convey mathematical meaning. In data from the whole year, there were significant differences between the youngest (the 1st age class) and the older 5th and 6th age classes for all analyzed elements, and between the 2nd and the 6th age class (with exception for $K^+$). Only a few significant differences were noticed between the 2nd and the 5th age class (for $NH_4^+$, $SO_4^{2-}$, $Mn^{2+}$, and $H^+$) and between the 1st and the 2nd age class (for $SO_4^{2-}$, $K^+$, $Ca^{2+}$, $Mg^{2+}$, $Mn^{2+}$). Between the 116- and the 91-year-old spruce stands, we noticed a significant difference only for $Mn^{2+}$. Taking into consideration the winter season, there was a significant difference between the youngest age class (1st) and the oldest (5th) and (6th), and between the 2nd age class and the oldest for all analyzed elements. Only a few significant differences were noticed between the 2nd and the 5th age class (for $NH_4^+$, $SO_4^{2-}$, $K^+$, $Ca^{2+}$, $Mg^{2+}$, $Mn^{2+}$) and between the 1st and the 2nd age class (for $SO_4^{2-}$, $K^+$, $Ca^{2+}$, $Mg^{2+}$, $Mn^{2+}$). Between the oldest spruce stands (5th and 6th age classes), we noticed a difference only for $Mn^{2+}$. In the vegetation period, there was a significant difference in the concentration of anions and cations between the youngest age class and the oldest (5th, with exception for $K^+$, and 6th), and the 2nd age class and the oldest (with exception for $NO_3^-$, $NH_4^+$, and $Mn^{2+}$). Only a few significant differences were noticed between the 2nd and the 5th age class (for $SO_4^{2-}$, $K^+$, $Mn^{2+}$, and $H^+$) and between the 1st and the 2nd age class (for $SO_4^{2-}$). Between the oldest spruce stands (5th and 6th age classes), there was a difference for $SO_4^{2-}$, $K^+$, $Ca^{2+}$, $Mg^{2+}$, and $Mn^{2+}$.

Detection and assessment of the capacity of the forest canopies of stands of different ages to modify ionic solutions as they move through the canopy was evaluated by PCA. The goal of the PCA is to achieve data projection, matrix dimensionality reduction (in sense of the monitoring dataset), and to offer a model for data interpretation. The factor layout obtained by PCA for both precipitation and throughfall solutions was similar for four stand age classes. It explained 67.60, 75.84, 74.34, 61.26, and 68.09% of the total variance system for BP and the 1st, 2nd, 5th, and 6th age classes, respectively (Table 3). Five significant factors were obtained for the 1st and 2nd age classes, four factors for BP and the 6th age class, and three in the case of the 5th age class. The factor conditionally named as “mineral dust”, referring to soil-dust particles, explains 27.71 (BP), 20.59 (1st), 15.95 (2nd), 17.38 (5th), and 32.30% (6th) of the total variance and was strongly correlated with $Na^+$, $K^+$, $Ca^{2+}$, and $Mg^{2+}$, especially in older age classes and BP. Apart from a lack of correlation between $K^+$ and $Ca^{2+}$ with other cations, the composition of the “mineral dust” factor is quite similar for the various forest stand age categories. This is in agreement with results obtained by others[25,26]. The factor conditionally named as “acidic emissions”, referring to coemission of $SO_4^{2-}$ and $NO_3^-$ precursors ($SO_2$, $NO_x$) associated with the combustion of fossil fuels, burning of biomass, and by automobile exhaust, explains 19.98 (BP), 26.72 (1st), 12.28 (2nd), and 34.03% (5th) of the total variance and was in most cases strongly correlated with $Cl^-$, $SO_4^{2-}$, and $NO_3^-$. In the case of the 5th age class category, this factor is supplemented by strongly correlated $NH_4^+$. The factor conditionally named as “heavy metals-dust particles”, referring to heavy metals connected with ash emissions, explains 8.48 (BP), 8.14 (1st), 28.76 (2nd), 34.03 (5th), and 16.64% (6th) of the total variance and includes strong correlations with $Fe^{2+}$, $Mn^{2+}$, and $Zn^{2+}$. The PC1 structure for the 5th category indicates an influence of

## TABLE 3
## Factor Loading and Explained Variance for BP and Four Categories of Throughfall Solutions Collected from the Norway Spruce (*P. abies* Karst) Forest Stands of Different Ages and Categories (1st, 2nd, 5th, 6th)* in the Dupniański Stream Catchment (1999–2003)

| | Precipitation | | | | Throughfall Solutions | | | | | | | | | | | | | | | | |
|---|---|---|---|---|---|---|---|---|---|---|---|---|---|---|---|---|---|---|---|---|---|
| | BP | | | | 1st | | | | | 2nd | | | | | 5th | | | 6th | | | |
| | PC1 | PC2 | PC3 | PC4 | PC2 | PC1 | PC3 | PC5 | PC4 | PC2 | PC3 | PC4 | PC1 | PC5 | PC2 | PC1 | PC3 | PC1 | PC4 | PC2 | PC3 |
| | ***PC1*** | ***PC2*** | ***PC3*** | ***PC4*** | ***PC1*** | ***PC2*** | ***PC3*** | ***PC4*** | ***PC5*** | ***PC1*** | ***PC2*** | ***PC3*** | ***PC4*** | ***PC5*** | ***PC1*** | ***PC2*** | ***PC5*** | ***PC1*** | ***PC2*** | ***PC4*** | ***PC5*** |
| V ($mm \cdot m^2$) | 0.18 | –0.57 | 0.04 | –0.34 | 0.18 | **–0.61** | 0.05 | –0.41 | 0.01 | –0.35 | –0.46 | –0.18 | –0.38 | –0.33 | –0.07 | **–0.71** | 0.30 | –0.11 | **–0.81** | –0.29 | 0.02 |
| $Cl^-$ ($mg \cdot l^{-1}$) | 0.21 | **0.72** | 0.06 | 0.12 | 0.28 | –0.02 | 0.70 | 0.39 | 0.26 | –0.17 | **0.87** | 0.24 | 0.01 | 0.10 | 0.18 | **0.69** | –0.10 | 0.03 | **0.61** | 0.15 | 0.55 |
| $NO_3^-$ ($mg \cdot l^{-1}$) | 0.11 | **0.86** | –0.08 | 0.11 | 0.04 | **0.81** | 0.01 | –0.04 | 0.29 | 0.12 | **0.87** | 0.01 | –0.01 | –0.08 | 0.24 | **0.68** | 0.25 | 0.42 | 0.41 | –0.30 | 0.39 |
| $NH_4^+$ ($mg \cdot l^{-1}$) | –0.17 | 0.23 | **0.81** | –0.19 | –0.22 | 0.12 | **0.91** | –0.15 | –0.12 | 0.10 | 0.27 | **0.64** | 0.48 | –0.22 | 0.02 | **0.75** | –0.24 | 0.29 | **0.71** | 0.03 | –0.16 |
| $SO_4^{2-}$ ($mg \cdot l^{-1}$) | 0.35 | **0.63** | 0.24 | 0.16 | 0.12 | **0.84** | 0.26 | 0.16 | 0.02 | 0.17 | **0.82** | 0.02 | 0.19 | 0.14 | 0.60 | **0.65** | 0.18 | 0.48 | 0.59 | 0.17 | 0.43 |
| $Na^+$ ($mg \cdot l^{-1}$) | **0.80** | –0.13 | –0.16 | –0.06 | **0.87** | –0.11 | –0.12 | –0.19 | 0.14 | 0.30 | 0.43 | 0.16 | **–0.69** | –0.09 | **0.71** | –0.18 | 0.18 | **0.63** | 0.04 | –0.26 | 0.40 |
| $K^+$ ($mg \cdot l^{-1}$) | **0.72** | 0.42 | 0.08 | –0.12 | 0.48 | 0.54 | 0.21 | 0.14 | –0.43 | **0.62** | 0.24 | 0.50 | –0.03 | 0.20 | **0.65** | 0.23 | –0.31 | **0.76** | 0.20 | 0.07 | –0.16 |
| $Ca^{2+}$ ($mg \cdot l^{-1}$) | **0.76** | 0.28 | 0.14 | 0.14 | 0.30 | 0.51 | –0.30 | –0.21 | –0.30 | **0.82** | 0.00 | –0.11 | 0.05 | –0.27 | **0.78** | 0.12 | 0.08 | **0.86** | 0.05 | 0.24 | 0.14 |
| $Mg^{2+}$ ($mg \cdot l^{-1}$) | **0.89** | 0.12 | 0.00 | –0.09 | **0.76** | 0.38 | 0.04 | –0.12 | –0.37 | **0.83** | 0.04 | 0.11 | –0.14 | 0.17 | **0.78** | 0.07 | –0.13 | **0.77** | 0.34 | –0.38 | 0.08 |
| $Fe^{2+}$ ($mg \cdot l^{-1}$) | 0.00 | 0.05 | –0.34 | **0.75** | –0.12 | –0.07 | –0.09 | **0.86** | 0.05 | –0.03 | 0.04 | –0.07 | 0.06 | **0.88** | –0.24 | **0.60** | 0.04 | –0.12 | 0.28 | **0.69** | –0.25 |
| $Mn^{2+}$ ($mg \cdot l^{-1}$) | –0.23 | 0.19 | 0.28 | **0.76** | 0.02 | 0.45 | 0.44 | **0.61** | –0.20 | 0.19 | 0.21 | 0.28 | **0.60** | 0.46 | 0.25 | **0.70** | –0.02 | 0.14 | –0.10 | **0.87** | 0.22 |
| $Zn^{2+}$ ($mg \cdot l^{-1}$) | 0.11 | 0.23 | –0.08 | **0.73** | –0.15 | 0.24 | 0.15 | **0.63** | 0.32 | –0.07 | 0.17 | –0.16 | **0.69** | 0.01 | –0.07 | **0.76** | 0.17 | –0.06 | 0.32 | **0.76** | 0.08 |
| $H^+$ ($\mu g \cdot l^{-1}$) | –0.43 | 0.32 | **–0.62** | –0.07 | –0.02 | 0.14 | 0.01 | 0.13 | **0.88** | –0.02 | –0.03 | **–0.84** | 0.26 | –0.03 | 0.00 | 0.00 | **0.92** | 0.04 | –0.07 | 0.06 | **0.81** |
| **Eigenvalue** | **3.01** | **2.51** | **1.36** | **1.91** | **2.68** | **3.47** | **1.44** | **1.06** | **1.21** | **2.07** | **1.59** | **1.18** | **3.74** | **1.08** | **2.26** | **4.42** | **1.28** | **4.20** | **1.15** | **2.55** | **1.34** |
| **Explained variance (%)** | **27.71** | **19.98** | **11.43** | **8.48** | **20.59** | **26.72** | **11.09** | **8.14** | **9.30** | **15.95** | **12.28** | **9.07** | **28.76** | **8.28** | **17.38** | **34.03** | **9.85** | **32.30** | **8.82** | **16.64** | **10.33** |
| **Cumulative explained variance (%)** | **27.71** | **47.69** | **59.12** | **67.60** | **20.59** | **47.31** | **58.40** | **66.54** | **75.84** | **15.95** | **28.23** | **37.30** | **66.06** | **74.34** | **17.38** | **51.41** | **61.26** | **32.30** | **41.12** | **57.76** | **68.09** |
| **Factors** | **PC1 – "mineral dust"<br>PC2 – "acidic emissions"<br>PC3 – "ammonium"<br>PC4 – "heavy metals-dust particles"** | | | | **PC1 – "mineral dust"<br>PC2 – "acidic emissions "<br>PC3 – "ammonium"<br>PC4 – "heavy metals-dust particles"** | | | | | **PC1 – "mineral dust"<br>PC2 – "acidic emissions"<br>PC3 – "ammonium"<br>PC4 – "heavy metals-dust particles"** | | | | | **PC1 – "mineral dust"<br>PC2 – "acidic emissions" + "heavy metals-dust particles"** | | | **PC1 – "mineral dust"<br>PC2 – "acidic emissions "<br>PC4– "heavy metals-dust particles"** | | | |

* The four stand age categories correspond to stands 11, 24, 91 and 116 years old.

Note: PC1, PC2, PC3, PC4, PC5 (regular) – primary notation connected with sequence of PCs obtained by PCA; *PC1, PC2, PC3, PC4, PC5 (italic) – changed notation to unify the factor identification.*

both "acidic emissions" and "heavy metals-dust particles" sources. For BP and the 1st and 2nd categories in the factor layout, there was a factor strongly influenced by $NH_4^+$ that explains 11.43, 11.09, and 9.07% of the total variance, respectively. The $NH_4^+$ present in the atmosphere might come from several sources including the volatilization of animal residues, human excrements, natural loss by plants, biomass burning, and industrial processes, such as the use or the production of fertilizers and emissions from the combustion of fossil fuels[27,28]. For the 5th and 6th categories, the influence of $NH_4^+$ is connected with variables belonging to the "acidic emissions" factor.

Concentration of major analytes classified as components of identified factors were summarized ("acidic emissions", $SO_4^{2-}$, $NO_3^-$; "heavy metals-dust particles", $Fe^{2+}$, $Mn^{2+}$, $Zn^{2+}$; "natural", $Na^+$, $K^+$, $Ca^{2+}$; "$NH_4^+$"; and $H^+$) and presented in Fig. 3, taking into account the division of throughfall into winter and the growing season (vertical lines represent ± S.D.). The particular coefficients of determination for winter season and all year can be summarized as follows: "acidic emissions" (winter: $R^2 = 0.99$, all: $R^2 = 0.91$); "heavy metals-dust particles" (winter: $R^2 = 0.72$, all: $R^2 = 0.54$); "mineral dust" (winter: $R^2 = 0.98$, all: $R^2 = 0.87$); "$H^+$" (winter: $R^2 = 0.97$, all: $R^2 = 0.93$); "$NH_4^+$" (winter: $R^2 = 0.81$, all: $R^2 = 0.91$). As the age of the spruce stands increased together with differences in canopy structure and leachability of foliage with a larger component of older foliage, the sum of anions of strong acids and concentrations of $H^+$

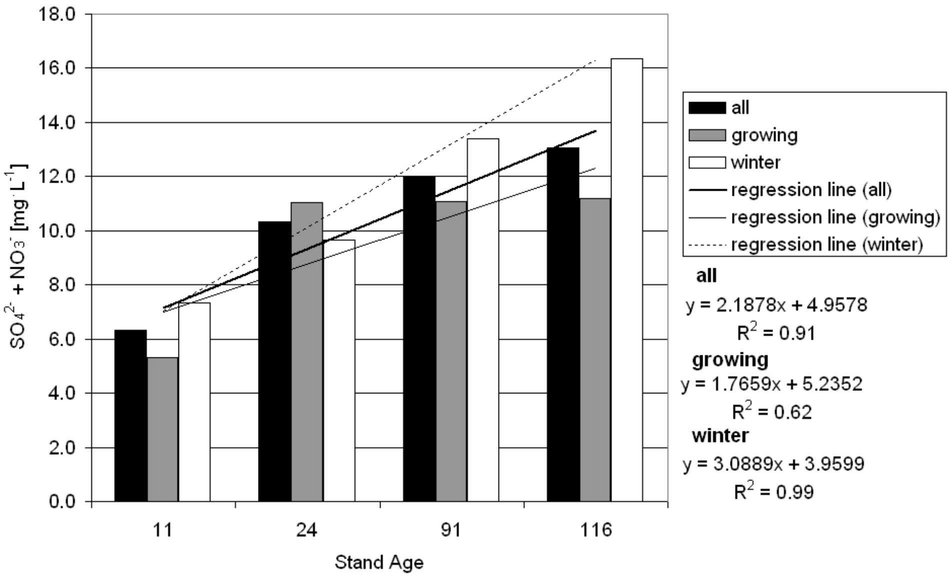

A

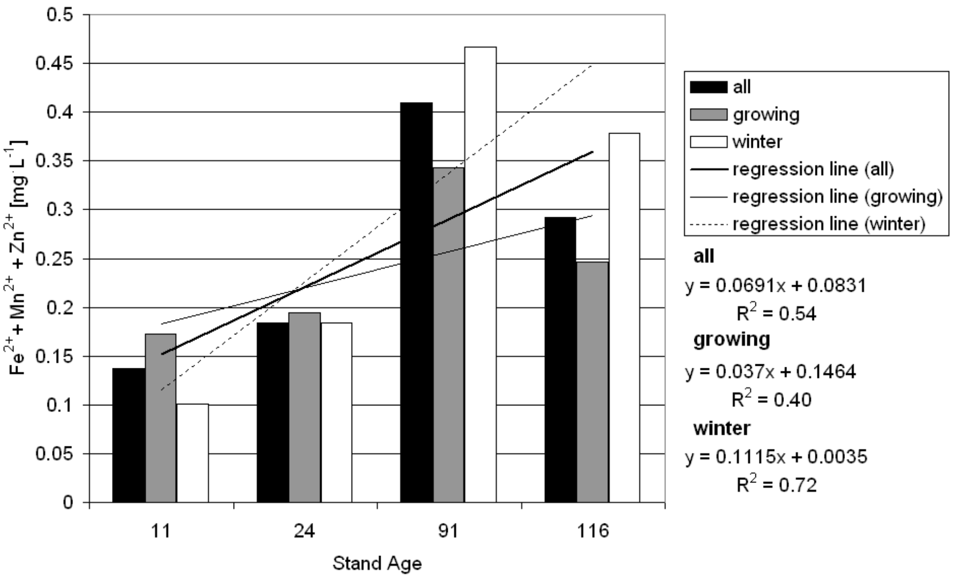

B

**FIGURE 3.** Linear regressions of (A) $\Sigma(SO_4^{2-} + NO_3^-)$, (B) $\Sigma(Fe^{2+} + Mn^{2+} + Zn^{2+})$, (C) $\Sigma(Na^+ + K^+ + Ca^{2+} + Mg^{2+})$, (D) $H^+$, (E) $NH_4^+$ in throughfall for four Norway spruce (*P. abies* Karst) stand age categories for the whole year, and during the winter and growing seasons.

increased over the whole year, and especially in winter. The same tendency was also noticed for the sum of cations and heavy metals in winter. During the growing season, this relationship was not so strong, probably because solutions were modified by processes occurring in the canopy (adsorption, leaching, etc.). Concentrations of $NH_4^+$ and $H^+$ increased with the age of spruce stands in winter and during the growing season. This is of particular concern because of likely increases in nitrification rates and soil acidification. Excess nitrogen and increasing N/cation ratios may have negative effects on the health condition of spruce stands[29]. A decrease in the pH of BP and throughfall solutions as a stand ages can have an effect on the soil neutralizing capacity[22].

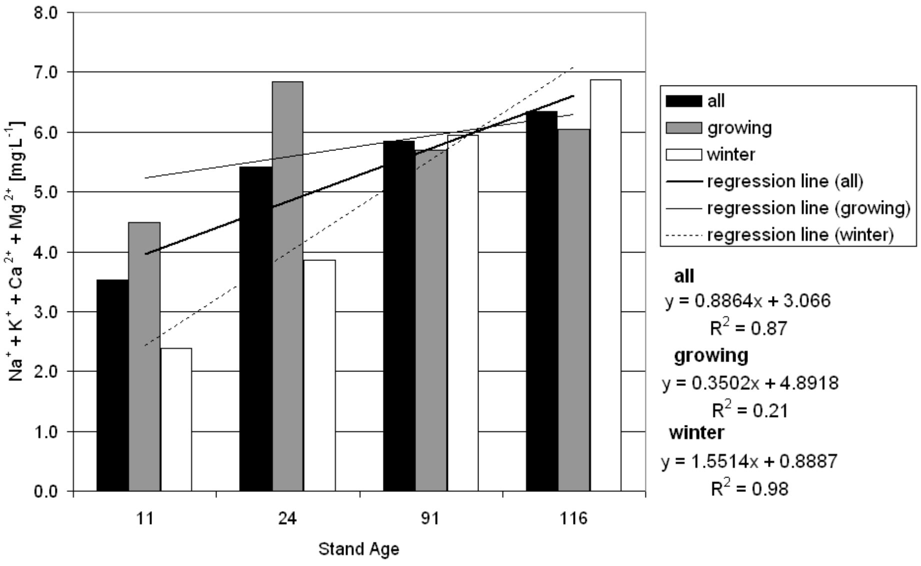

FIGURE 3C

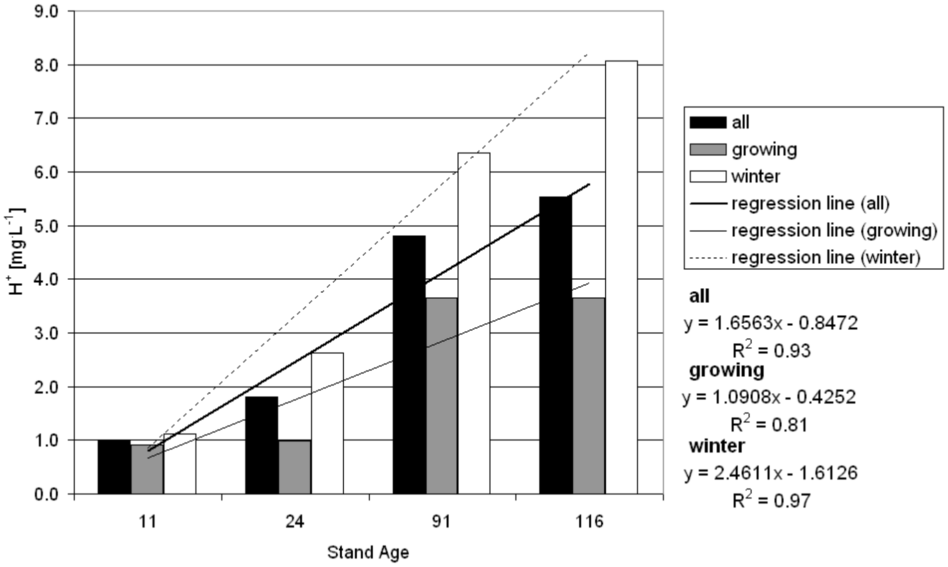

FIGURE 3D

## CONCLUSION

1. The chemical composition of throughfall solutions depends on the age of the spruce stands and season of the year. A decrease in the pH of throughfall and an increase in the amount of hydrogen ion deposited to the soil in throughfall with increasing stand age were especially noted in the winter season.
2. Concentrations in throughfall of $K^+$, $H^+$, $SO_4^{2-}$, $Mn^{2+}$,and $NH_4^+$ were greater than in BP over the whole year, and in winter and the growing seasons, concentrations of $K^+$, $H^+$, and $Mn^{2+}$ were greater in throughfall. This suggests that these ions were washed out or washed from the surface of needles and/or the bark. The other ions $NO_3^-$, $NH_4^+$, $Ca^{2+}$, $Mg^{2+}$ (especially in vegetation period), $Fe^{2+}$, and $Zn^{2+}$ were absorbed in the canopy, especially in older Norway spruce stands.

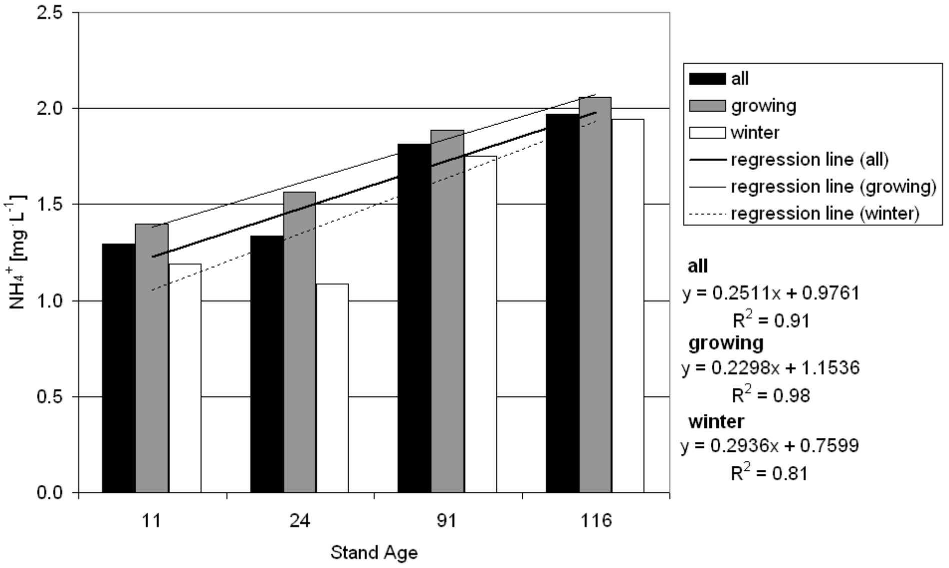

**FIGURE 3E**

3. Nitrogen deposition was above critical loads for coniferous trees, which may change N/macronutrient ratios, decrease K and Mg, and increase N concentration in foliar tissue, and which may be an increasing problem in the future unless emissions of nitrogen oxides, and especially $NH_4^+$, are reduced.
4. A 5-year throughfall study on the Dupniański catchment indicated an increasing amount of sulfur and of the sum of the strong acids (S-$SO_4^{2-}$ and N-$NO_3^-$) deposited to the soil, especially in older spruce age classes, which may have implications for the vitality of spruce stands.
5. The application of PCA has led to identification in general of five factors responsible for the data structure (“mineral dust”, “acidic emissions”, “heavy metals-dust particles”, “$NH_4^+$”, and “$H^+$”). They explain 61–76% of the total variance system for BP and the 1$^{st}$, 2$^{nd}$, 5$^{th}$, and 6$^{th}$ spruce age classes. The percentage of variance explained refers not to some definitive environmental events, but to the validity of the multivariate statistical model, which tries to indicate possible sources of the deposited ions.
6. The strong positive correlation between spruce age classes and ionic concentrations in throughfall occurs for all year and the winter period for ions within the following categories: “acidic emissions”: $SO_4^{2-}$ + $NO_3^-$; “heavy metals-dust particles”: $Fe^{2+}$ + $Mn^{2+}$ + $Zn^{2+}$; “mineral dust”: $Na^+$ + $K^+$ + $Ca^{2+}$ + $Mg^{2+}$; “$NH_4^+$”; and “$H^+$”. The strength of the relationship decreases in the growing period, probably due to the processes occurring in the canopy (adsorption, leaching, etc.).
